# Association between polymorphism at IGF-1 rs35767 gene locus and long-term decline in renal function: a Japanese retrospective longitudinal cohort study

**DOI:** 10.1186/s12882-021-02408-9

**Published:** 2021-06-02

**Authors:** Kosuke Honda, Satoru Kuriyama, Kimiyoshi Ichida, Tomoko Nakano, Naoki Sugano, Takashi Yokoo

**Affiliations:** 1 Division of Nephrology and Hypertension, Department of Internal Medicine, The Jikei University School of Medicine, Tokyo, Japan; 2 Nephrology & Hypertension Research Unit, Miho Clinic, Tokyo, Japan; 3 Department of Pathophysiology, Tokyo University of Pharmacy and Life Sciences, Tokyo, Japan; 4 Health Management Center of the Tokyo Regional Taxation Bureau Clinic, Tokyo, Japan

**Keywords:** IGF-1, Gene polymorphism, Blood pressure, Glomerular filtration rate

## Abstract

**Background:**

Insulin-like growth factor-1 (IGF-1) acts on glucose and protein metabolism and human growth and also influences blood pressure and renal function. This study investigated whether the single-nucleotide polymorphism of IGF-1, rs35767, plays a role in metabolic syndrome indicators, including blood pressure, glucose metabolism, uric acid levels, and renal function.

**Methods:**

In this retrospective longitudinal cohort study, blood samples from 1506 Japanese individuals were collected and used for genotyping for variant rs35767: T > C in the IGF-1 upstream promoter. Data were analyzed to identify associations between IGF-1 genotypes and patient biochemical parameters, including the components of metabolic syndrome and the long-term change in renal function.

**Results:**

The cohort rs35767 genotypes included 650 CC carriers (43.2%), 687 TC carriers (45.6%), and 169 TT carriers (11.2%). Multiple regression analysis revealed no association between IGF-1 genotype and blood pressure, glycated hemoglobin level, and serum uric acid level. However, in females, blood pressure was negatively correlated with the TT genotype. Longitudinal observation revealed that the decline in eGFR over 10 years was greater in TT (− 18.51 ± 1.04 mL/min/1.73m^2^) than in CC carriers (− 16.38 ± 0.52 mL/min/1.73m^2^; *P* < 0.05).

**Conclusion:**

The present study suggests that renal function declines faster in individuals with the TT genotype at the IGF-1 rs35767 locus than in those with the CC genotype, suggesting that the TT genotype is associated with the long-term chronological decline in renal function.

## Background

Insulin-like growth factor-1 (IGF-1), also known as somatomedin C, is an endocrine hormone produced in the liver. IGF-1 exerts insulin-like action, affecting human growth and cell proliferation. The secretion of IGF-1 is stimulated by growth hormone, insulin, and dietary protein intake and is modulated by age, sex, ethnicity, and genetic predisposition. IGF-1 not only acts on glucose and protein metabolism but also has an influence on blood pressure (BP) and renal function. Circulating IGF-1 normally reaches peak levels during puberty, gradually declining with advancing age [1]. The role of IGF-1 in BP regulation is complex. Studies have shown that IGF-1 levels are higher in patients with hypertension than in those without [2–6]. In patients with excessively high IGF-1 levels, as in acromegaly, a positive relationship has been observed between IGF-1 level and BP [7]. However, other studies report that the relationship between BP and IGF-1 level is neutral [8, 9] or even inverse [10–18]. In an animal model, IGF-1 was reported to decrease systemic BP and increase blood flow in selective vascular beds [19]. In addition, IGF-1 directly stimulates nitric oxide (NO) production in endothelial cells [20]. Interestingly, in spontaneously hypertensive rats, IGF-1-induced vasorelaxant effects are impaired [21]. In contrast, GH and IGF-1 act on the renal tubule to promote the retention of sodium and water in the body, contributing to sodium-dependent hypertension [22]. Together, these observations suggest that IGF-1 helps in either increasing or lowering BP, depending on the conditions.

Genome-wide association studies show that the total number of hypertension-related gene loci is well over 500, even if it is limited to common mutations among determined genes. Although their influence on BP in the general population is only about 1 mmHg [23], some gene mutations are reported to have a relatively large effect on BP. Most of these mutations affect water/electrolyte channels and transporters at the tubular level [24, 25].

Clinical evidence suggests that the T/C polymorphism rs35767 near the promoter region of the IGF-1 gene is associated with plasma IGF-1 levels. IGF-1 levels are higher in T carriers than in CC carriers. Interestingly, another study suggests that IGF-1 plays a role in the pathogenesis of atherosclerosis [26]. In addition, single-nucleotide polymorphism analysis of IGF-1 showed that rs35767 is associated with hypertension in Europeans [5]. A study of the relationship between IGF-1 polymorphism rs35767 and serum uric acid (UA) levels showed that non-Asian CC carriers have elevated UA levels [27]. Despite this preliminary evidence that IGF-1 plays a role in BP regulation, studies investigating the role of IGF-1 in renal function are limited.

This study investigates whether the IGF-1 gene polymorphism rs35767 is associated with metabolic syndrome indicators, including BP and glycated hemoglobin (HbA1c). Of particular focus is the relationship between IGF-1 genotype and long-term changes in renal function.

## Methods

### Aim, design, and setting

This study aimed to investigate whether the IGF-1 gene polymorphism rs35767 is associated with metabolic syndrome indicators, including BP and glycated hemoglobin (HbA1c). This is a retrospective, longitudinal cohort study conducted in Japan.

### Characteristics of participants

**Fig. 1:**
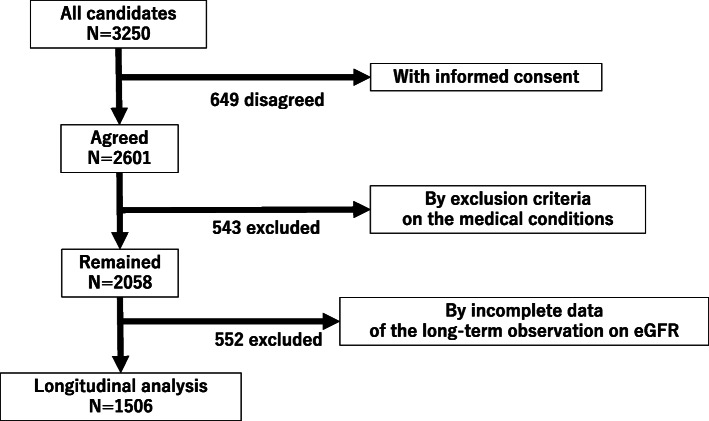
A flow diagram of the study. A total of 3250 candidates were asked to participate in the study with the written informed consent with an invitation letter, in which 2601 individuals agreed. After applying exclusion criteria on medical conditions such as hypertension, diabetes mellitus and hyperuricemia during the allocated period, a total of 543 were excluded, and 2058 remained. In addition, a total of 552 were also excluded due to incomplete data on long-term observation on eGFR. The final number for the longitudinal analysis was 1506

The original candidate group included 3250 individuals, all native Japanese living in the vicinity of Tokyo. They attended annual medical check-ups at our health management center. As a result, 2601 of the 3250 individuals agreed to participate in the study. Of the 2601 patients, some [*n* = 543, 20.9%] were excluded from the study, because they were treated for medical conditions, including hypertension [*n* = 283 (10.9%); F/M, 11/272], diabetes mellitus [*n* = 111 (4.3%); F/M, 4/107], and hyperuricemia [*n* = 149 (5.7%); F/M, 0/149] during the allocated period. Individuals whose renal function could not be monitored for a period of 10 years were also excluded [*n* = 552]. The final analysis was then made using 1506 individuals. The flow diagram of the study was shown in Fig. 1.

### Laboratory tests

Blood was drawn after an 8–12-h fasting. Measured biochemical parameters included serum aspartate aminotransferase (AST), alanine aminotransferase (ALT), creatinine (Cr), and UA levels, as well as lipid profiles of total cholesterol, high-density lipoprotein cholesterol (HDLC), low-density lipoprotein cholesterol (LDLC), triglyceride (TG), and HbA1c.

### Other variables

Body mass index (BMI) was calculated using the following equation: BMI = Body weight × 1/(body height)^2^. BP was measured with the patient in a sitting position during a morning visit (fasting state, time: 9–11 AM) after 5 min of rest in a supine position (to avoid the so-called “white coat hypertension”) using an automatic self-measuring device equipped with a 47 × 13-cm cuff and 24 × 13-cm bladder. Mean BP (MBP) was calculated from systolic BP (SBP) and diastolic BP (DBP) using the following equation: MBP = DBP + (SBP − DBP)/3. For the Japanese individuals, renal function expressed as eGFR was calculated using the following equation: eGFR = 194 × Cr^− 1.094^ × age^− 0.287^(for women, × 0.739), as reported elsewhere [28]. Laboratory tests were performed using the BioMajesty auto-analyzer Series JCA-9130 (JOEL, Tokyo, Japan).

### DNA analysis

Genomic DNA was extracted from whole peripheral blood cells, and samples were stored at − 80 °C until use. Polymorphism analysis was performed by Sanger sequencing [29]. PCR reactions were performed in a total volume of 20 μL containing 50 ng genomic DNA, TaKaRa Ex Taq DNA Polymerase supplied by Takara Bio Inc., and 10 μM of each forward and reverse primers. PCR amplification was performed in a DNA thermal cycler (BIO-RAD DNA Engine Peltier Thermal Cycler). The amplification conditions were as follows: initial denaturation at 94 °C for 5 min followed by 35 cycles of 94 °C for 30 s, 60 °C for 30 s, and 72 °C for 30 s, with final extension for 5 min at 72 °C. The amplified PCR products were visualized by 1% agarose gel electrophoresis under UV light. Primers ordered from Eurofins Genomics K.K. Tokyo, Japan, were as follows; 5′-TTGGGCACATAGTAGAGCTCAC-3′ and 5′-CAAAAGCCCAGAGCAGACAT-3′.

### Statistical analysis

The database and all statistical outputs were retained by the University. Access to the database was limited as deemed necessary. The authors assume full responsibility for the completeness and accuracy of the content of the manuscript. Results of the different subgroups were examined by one-way analysis of variance. The final variables were chosen on the basis of clinical importance and biological plausibility at the investigators’ discretion. Multiple regression analysis was used to calculate the regression coefficient (β) and standard error (SE) to estimate factors affecting BP, HbA1c, UA, and eGFR after adjusting confounders. For the evaluation of long-term changes in eGFR (ΔeGFR), longitudinal analyses were performed. For the further evaluation of the age effect, participants were divided into the younger age [18-32y.o., *n* = 768, F/M = 168/600] and the older age [33-55y.o., *n* = 738, F/M = 38/700].

Statistical analyses were performed using Stat Flex version 7.0 (Artec Ltd. Co., Osaka, Japan) and EZR (Version 1.33, Saitama Medical Center, Jichi Medical University, Saitama, Japan), which is a graphical user interface for R (The R Foundation for Statistical Computing, Vienna, Austria). EZR is a modified version of R Commander designed to add statistical functions frequently used in biostatistics. Data are presented as the mean ± standard deviation unless otherwise indicated. *P*-values of ≤0.05 were considered statistically significant. Because the histograms of each parameter distributed in a parametric manner, logarithmic transformation was not performed.

## Results

The final cohort included 206 females and 1300 males [*n* = 1506; 13.8% female]. The cohort was grouped according to IGF-1 rs35767 genotype as CC [*n* = 650 (43.2%); F/M, 101/549], TC [*n* = 687 (45.6%); F/M, 83/604], and TT [*n* = 169 (11.2%); F/M, 22/147] (Table 1).

**Table 1:** Characteristics of participants according to the SNP rs35767 genotype^a^

	Genotype			
	CC	TC	TT	*P*
Sex (Female/Male)	101/549	83/604	22/147	0.17
Age (years)	33.8 ± 9.4	34.0 ± 9.3	33.8 ± 8.7	0.89
BMI (kg/m^2^)	22.9 ± 3.6	22.9 ± 3.5	22.7 ± 3.2	0.76
SBP (mmHg)	126.6 ± 17.1	127.6 ± 16.5	126.2 ± 17.5	0.42
DBP (mmHg)	76.6 ± 11.8	77.6 ± 11.4	76.2 ± 12.2	0.22
MBP (mmHg)	93.28 ± 12.5	94.2 ± 12.1	92.9 ± 13.2	0.25
AST (IU/L)	22.9 ± 11.4	23.5 ± 11.3	23.4 ± 10.1	0.68
ALT (IU/L)	26.1 ± 21.6	26.4 ± 19.9	25.2 ± 17.9	0.67
Cr (mg/dL)	0.77 ± 0.18	0.77 ± 0.23	0.78 ± 0.28	0.87
eGFR (mL/min/1.73m^2^)	96.8 ± 19.8	96.8 ± 20.1	98.3 ± 19.4	0.65
UA (mg/dL)	5.8 ± 1.4	5.8 ± 1.4	5.9 ± 1.4	0.81
TC (mg/dL)	195.1 ± 33.1	194.8 ± 32.9	193.6 ± 31.8	0.27
HDLC (mg/dL)	65.8 ± 16.3	66.0 ± 16.1	64.9 ± 15.3	0.83
LDLC (mg/dL)	116.0 ± 31.6	112.6 ± 30.0	112.4 ± 28.1	0.10
TG (mg/dL)	104.1 ± 80.2	106.9 ± 82.8	99.9 ± 81.9	0.27
HbA1c (%)	5.32 ± 0.59	5.32 ± 0.68	5.20 ± 0.45	0.07

^a^*n* = 1506

Data are presented as the mean ± standard deviation

Abbreviations: *SNP* single-nucleotide polymorphism, *IGF-1* insulin-like growth factor-1, *BMI* body mass index, *SBP* systolic blood pressure, *DBP* diastolic blood pressure, *MBP* mean blood pressure, *AST* aspartate transaminase, *ALT* alanine transaminase, *Cr* creatinine, *eGFR* estimated glomerular filtration rate, *UA* uric acid, *TC* total cholesterol, *HDLC* high-density lipoprotein cholesterol, *LDLC* low-density lipoprotein cholesterol, *TG* triglyceride, *HbA1c* glycated hemoglobin

Biochemical parameters of participants according to rs35767 genotype are shown in Table 1. No difference was found in any of the parameters including SBP, DBP, MBP, serum Cr concentration, eGFR, serum UA level, HbA1c among the three groups.

The association between IGF-1 genotype and BP (SBP, DBP, and MBP) was determined using multiple regression analysis with CC as the standard for comparison (Table 2). Using three models (Model 1, male only; Model 2, female only; and Model 3, both sexes), In any of the three independent models (Model 1, 2 and 3), we observed that SBP, DBP and MBP were all associated with the TT genotype (*P* < 0.05) in females, but not in male. In contrast, no association was observed between rs35767 genotype and HbA1c level, serum UA level, or eGFR in any of the three models (Table 3).

**Table 2:** Association of rs35767 genotype with BP according to sex

	SBP						DBP						MBP					
Model	1(M)		2(F)		3(All)		1(M)		2(F)		3(All)		1(M)		2(F)		3(All)	
	β (SE)	*p*	β (SE)	*p*	β (SE)	*p*	β (SE)	*p*	β (SE)	*p*	β (SE)	*p*	β (SE)	*p*	β (SE)	*p*	β (SE)	*p*
CC	Ref		Ref		Ref		Ref		Ref		Ref		Ref		Ref		Ref	
TC	0.89 (0.92)	0.33	0.55 (1.91)	0.77	1.06 (0.84)	0.20	0.24 (0.60)	0.38	− 0.97 (1.61)	0.55	0.91 (0.56)	0.10	1.12 (0.64)	0.08	−0.46 (1.57)	0.41	0.96 (0.59)	0.10
TT	0.60 (1.44)	0.68	−5.96 (2.95)	<0.05	−0.02 (1.32)	0.99	0.69 (0.94)	0.46	−5.61 (2.50)	< 0.05	−0.21 (0.88)	0.81	0.66 (1.01)	0.51	−5.72 (2.43)	< 0.05	−0.14 (0.94)	0.88

Model 1, males only (M); Model 2, females only (F); Model 3, males and females (All); all models adjusted for age, BMI, eGFR, UA, LDLC, HbA1c

*n* = 1506 (F/M, 206/1300)

*SBP* systolic blood pressure, *DBP* diastolic blood pressure, *MBP* mean blood pressure, *BMI* body mass index, *eGFR* estimated glomerular filtration rate, *UA* uric acid, *LDLC* low-density lipoprotein cholesterol, *HbA1c* glycated hemoglobin, *β* regression coefficients, *ref*. reference, *SE* standard error

**Table 3:** Association of rs35767 genotype with HbA1c, UA, and eGFR according to sex

	HbA1c						UA						eGFR					
Model	1(M)		2(F)		3(All)		1(M)		2(F)		3(All)		1(M)		2(F)		3(All)	
	β (SE)	*p*	β (SE)	*p*	β (SE)	*p*	β (SE)	*p*	β (SE)	*p*	β (SE)	*p*	β (SE)	*p*	β (SE)	*p*	β (SE)	*p*
CC	Ref		Ref		Ref		Ref		Ref		Ref		Ref		Ref		Ref	
TC	−0.01 (0.02)	0.89	0.01 (0.05)	0.95	− 0.01 (0.02)	0.86	−0.03 (0.07)	0.65	0.02 (0.12)	0.89	0.03 (0.06)	0.61	0.19 (0.86)	0.83	1.67 (3.00)	0.58	0.29 (0.86)	0.73
TT	−0.05 (0.03)	0.13	−0.09 (0.07)	0.19	−0.06 (0.03)	0.05	0.05 (0.10)	0.61	0.04 (0.19)	0.81	0.10 (0.10)	0.32	1.99 (1.35)	0.14	1.70 (4.71)	0.72	2.06 (1.35)	0.12

Model 1, males only (M); Model 2, females only (F); Model 3, males and females (All); all models adjusted for age, BMI, MBP, eGFR, UA, LDLC, HbA1c

*n* = 1506 (F/M, 206/1300)

Abbreviations: *HbA1c* glycated hemoglobin, *UA* uric acid, *eGFR* estimated glomerular filtration rate, *BMI* body mass index, *LDLC* low-density lipoprotein cholesterol, *MBP* mean blood pressure, *β* regression coefficients, *ref*. reference, *SE* standard error

The results of longitudinal analysis of factors to explain time-dependent ΔeGFR are shown in Table 4. In a total of 1506 participants whose renal function was monitored for a period of 10 years, we observed a gradual decline in eGFR of approximately 1.0–1.5 mL/min/1.73m^2^/year. A significant negative correlation was observed between ΔeGFR and MBP (*P* < 0.05), and between ΔeGFR and HbA1c (*P* < 0.05). ΔeGFR correlated positively with serum UA level (*P* < 0.05). Notably, a negative association was observed between ΔeGFR and the TT genotype (*P* < 0.05). With respect to the effect of age on ΔeGFR, participants were divided into two groups; the younger and the older age. The negative association between ΔeGFR and the TT genotype remained significant in the younger age, while it became insignificant in the older age (Table 4).

**Table 4:** Longitudinal analysis of factors to explain the decline in eGFR^a^

Objective variable	⊿eGFR in 10 years								
	All			Younger age			Older age		
	β (SE)	t value	*p* value	β (SE)	t value	*p* value	β (SE)	t value	*p* value
BMI	−0.01 (0.11)	−0.08	0.94	−0.17 (0.17)	−0.98	0.32	0.01 (0.15)	0.05	0.96
MBP	−0.06 (0.03)	−1.97	< 0.05	−0.06 (0.05)	−1.26	0.20	−0.05 (0.04)	−1.36	0.18
UA	1.17 (0.28)	4.12	< 0.05	1.69 (0.44)	3.84	< 0.05	0.59 (0.37)	1.60	0.11
HbA1c	−2.30 (0.57)	−4.07	< 0.05	2.22 (1.77)	1.25	0.21	−2.83 (0.60)	−4.70	< 0.05
rs35767 polymorphism									
CC	Ref			Ref			Ref		
TC	−0.19 (0.67)	−0.28	0.78	−0.99 (0.96)	−1.03	0.31	0.71 (0.93)	0.77	0.44
TT	−2.42 (1.06)	−2.28	< 0.05	−3.90 (1.53)	−2.55	< 0.05	−1.02 (1.47)	−0.70	0.49

^a^*n* = 1506 individuals who were followed up for 10 years (F/M = 206/1300)

Younger age (18-32y.o.), *n* = 768 (F/M 168/600)

Older age (33-55y.o.), *n* = 738 (F/M = 38/700)

Model includes sex, age, BMI, MBP, UA, HbA1c, and rs35767 genotype

**Fig. 2:**
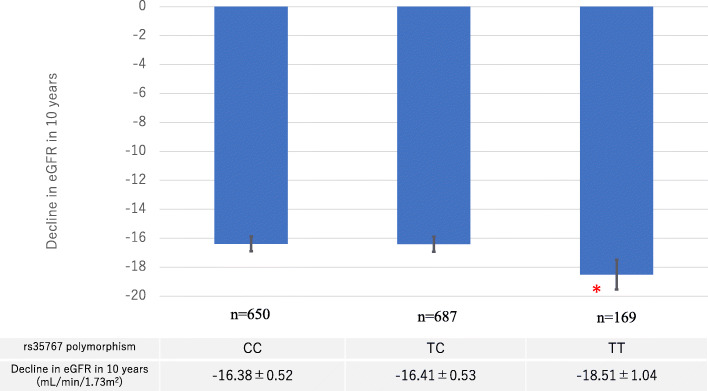
Decline in eGFR according to three IGF-1 rs35767 genotypes. *n* = 1506, (F/M = 206/1300) individuals who were followed up for 10 years. *P < 0.01, by multiple regression analysis. Bars represent standard error of the mean

The decline in eGFR over 10 years according to genotype is shown in Fig. 2. eGFR declined significantly faster in TT carriers (18.51 ± 1.04 mL/min/1.73m^2^; *n* = 169) than in CC carriers (16.38 ± 0.52 mL/min/1.73m^2^; *n* = 650) (*P* < 0.05).

## Discussion

The notable finding of this study is that the TT genotype of the rs35767 IGF-1 gene polymorphism is associated with a faster decline in eGFR than the CC genotype. This finding suggests that IGF-1 is not merely a metabolic growth hormone but may also influence BP and long-term renal function.

eGFR normally declines with age by approximately 1.03 mL/min/1.73m^2^/year, even in the absence of progressive renal diseases such as moderate-to-severe hypertension, overt diabetic nephropathy, and primary glomerular disease [30]. This age-related decline in renal function is caused by changes in renal morphology resulting from arteriosclerosis and concomitant renal atrophic changes. Renal function is greatly influenced by inner and middle membrane thickening and luminal narrowing that occur with age. The resulting decrease in glomerular blood flow causes collapse of the glomerular tuft and ischemia in the glomeruli and interstitial tissue, leading to an irreversible gradual loss of renal function known as nephrosclerosis.

Despite the observed association between the TT genotype and accelerated eGFR decline, BP in females were negatively associated with the TT genotype (Table 2). The reason for this contradiction is unclear. The negative association between TT genotype and BP suggests that the TT genotype has either a BP-lowering or a renal-protective effect. The association of BP with the TT genotype was observed only in females; thus, we believe that this discrepancy is probably attributable to the relatively small number of female participants (15.9%); the benefits of lowering BP on eGFR may have been offset by the majority of male participants.

The serum UA levels were comparable among the three genotypes (Table 1). Our observation is not in accordance with a previous report showing that serum UA levels are lowest in TT carriers in a non-Asian, sex-matched study [27]; TT carriers also displayed higher uricosuria levels than CC carriers. Our differing result may stem from differences in the study population, including ethnicity (non-Asians vs Asians in our study), differences in sex distribution (well-balanced sex ratio vs. small number of females in our study), and age distribution (old age vs. relatively younger age in our study).

Regarding the relationship between UA and eGFR, the longitudinal study showed a positive correlation between these parameters (Table 5). The physiological role of UA as an oxidant is supported by a number of in vitro and in vivo studies reporting that intracellular UA causes inflammation, oxidative stress, endothelial dysfunction, and activation of the renin–angiotensin–aldosterone system [31]. However, UA is also known as a powerful antioxidant in the extracellular milieu [32]. Importantly, numerous studies have shown that UA is a risk factor for the progression of hypertension and CKD. For example, Kamei et al. reported that a slight increase in serum UA level within the normal range causes a decrease in renal function [33]. Conversely, in individuals with mild-to-moderate renal disease, UA is not associated with elevated serum Cr level or end-stage renal disease adjusted for eGFR and proteinuria [34]. The effect of UA on renal function may not be renal-toxic unless serum UA levels are extremely high [35]. At an early stage, renal dysfunction caused by hyperuricemia is predominantly arteriosclerosis-related; with progression, a J-shaped phenomenon occurs between serum UA level and intraglomerular pressure, with a concomitant increase in renal afferent arteriolar resistance [36]. In the present study, serum UA levels were nearly within normal range (approximately 5.0–6.5 mg/dL) (Table 1). Despite this condition, the deleterious effect of UA on renal function was observed here.

Previous studies have shown that serum IGF-1 level depends on genotype, with TT > TC > CC [37, 38]. Accordingly, the observed decline in renal function could result from the constantly elevated IGF-1 levels. IGF-1 given intravenously decreases systemic BP and increases blood flow in selective vascular beds in animal model [19]. In addition, IGF-1 directly stimulates NO in endothelial cells [20], leading to an increase in glomerular blood flow [39]. However, GH and IGF-1 also induce hypertension. Indeed, studies show that patients with hypertension have higher IGF-1 levels than those without [2–6]. Furthermore, in a literature review of 20 studies including 11,704 subjects, Schutte et al. found a positive relationship between IGF-1 levels and BP for high IGF-1 levels in conditions such as acromegaly [7]. Notably, impairment of the IGF-1-induced vasorelaxant effects has been reported in spontaneously hypertensive rats [21]. Together, these observations indicate that IGF-1 increases BP under most circumstances but can lower BP in certain medical conditions. In the presence of excessive salt, individuals with TT genotype with elevated serum IGF-1 level might be susceptible to hypertension and CKD. We speculate that presumably elevated IGF-1 levels in TT carriers resulted in their faster decline in renal function over 10 years.

IGF-1 is a hormone that decreases with age [40]. We addressed the effect of age on the TT-related decline in eGFR by comparing the younger participants with the older ones. The faster decline in eGFR in TT carriers in the younger age remained significant compared with that in the older age. This could be explained by the fact that IGF-1 levels might have been higher in the younger age. The decline may be related to the age-related other factors such as salt sensitivity, BP, and NO production. While gaining insight into the mechanism underlying the faster decline in eGFR in TT carriers is important, such work is beyond the scope of this study.

Although this study is unique in its relatively large cohort (1506 individuals) and the long follow-up period on renal function (10 years), there are some limitations. First, all of the participants lived in the vicinity of Tokyo and were of Japanese ethnicity. So, extrapolation of the result to other ethnic groups is not always justified. Second, the low proportion of female participants may be problematic. Third, serum IGF-1 level was not measured. Modification of such innate problems would have increased the strength of our findings.

## Conclusions

This study provides preliminary evidence that the TT genotype at rs35767 in the IGF-1 gene is associated with long-term chronological decline in renal function. Future study is needed to clarify the relationship between rs35767 and renal function.

## Data Availability

The datasets used and/or analyzed in this study are available on reasonable request from the corresponding author.

## Abbreviations

ALT: Alanine transaminase
AST: Aspartate transaminase
BMI: Body mass index
BP: Blood pressure
Cr: Creatinine
DBP: Diastolic blood pressure
eGFR: Estimated glomerular filtration rate
HbA1c: Glycated hemoglobin
HDLC: High-density lipoprotein cholesterol
IGF-1: Insulin-like growth factor-1
LDLC: Low-density lipoprotein cholesterol
MBP: Mean blood pressure
SBP: Systolic blood pressure
TG: Triglyceride
UA: Uric acid
